# Acute toxicity of an organophosphate insecticide sumithion to striped catfish *Pangasianodon hypophthalmus*

**DOI:** 10.1016/j.toxrep.2019.09.004

**Published:** 2019-09-17

**Authors:** S. M. Majharul Islam, Md. Atiqur Rahman, Sadiqun Nahar, Md. Helal Uddin, Md. Mahfuzul Haque, Md. Shahjahan

**Affiliations:** Laboratory of Fish Ecophysiology, Department of Fisheries Management, Bangladesh Agricultural University, Mymensingh, 2202, Bangladesh

**Keywords:** Organophosphate insecticide, Hematological parameters, Blood glucose, Erythrocytes, Thai pangas

## Abstract

Sumithion is widely used for crop safety and eradication of tiger bugs (*Cicindela* spp.) from larval rearing aquaculture ponds. To satisfy the high demand of fries and fingerlings of widely cultured species striped catfish, spawns are produced in large scale in the hatcheries through hormone induced breeding, and subsequently these spawns are reared in nursery ponds and marketed to fingerlings vendors for stocking in grow-out ponds. Considering the importance of healthy fries and fingerlings the present experiment was conducted to evaluate the toxic effects of sumithion on striped catfish fingerlings. Fish were exposed for 96 h to six concentrations of sumithion (0, 3, 4, 5, 6 and 7 mg/l) each with three replications. The 96 h LC50 value was calculated using probit analysis. After 96 h of exposure fishes were sacrificed to measure hemato-biochemical (Hemoglobin, Hb; Red blood cell, RBC; White blood cell, WBC and blood glucose) parameters. In addition, formation of micronucleus (MN) was examined in the blood erythrocytes. The 96 h LC50 value of sumithion for striped catfish was 5.886 mg/l. The values of RBCs and Hb decreased significantly in different concentrations of the toxicant compared to control, while the values of WBC and blood glucose levels showed opposite scenario. Consequently, the frequencies of formation of MN increased significantly in different concentrations of the toxicant compared to the control. The results of the current study denoted that sumithion exerts toxicity to striped catfish. It is expected that the findings of the present research will help in the development of awareness of the concerned people about the toxic effect of sumithion as well as other insecticides and pesticides in the environment.

## Highlights

• The the median lethal concentration (96 h LC50) value of sumithion for striped catfish was 5.886 mg/l.
• The values of RBCs and Hb decreased significantly in different concentrations of sumithion.
• The values of WBC and blood glucose levels increased significantly in different concentrations of sumithion.
• Frequencies of formation of micronucleus (MN) were elevated by sumithion.

## Graphical abstract

**Figure:**
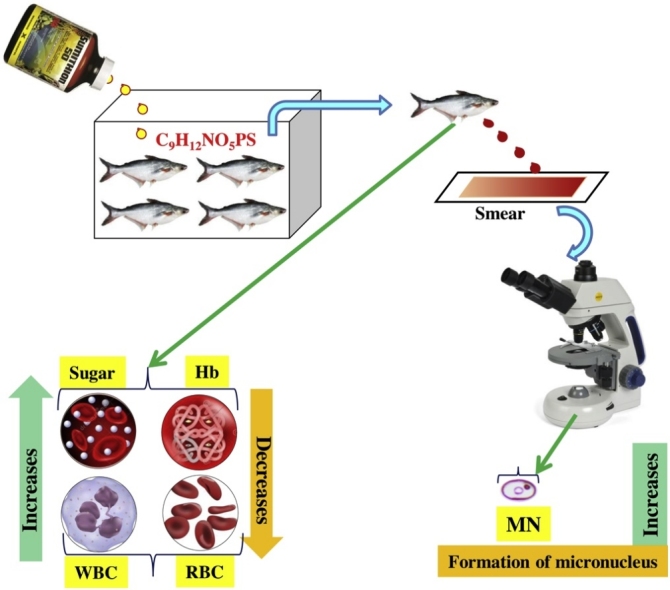

## 1 Introduction

Bangladesh is an agrarian country and its economy mainly depends on agricultural crop production. This principal argo-production sector is frequently invaded by pests and parasites, causing a severe economic decrement. To salvage from these worse situations, several types of remedial measures have been promoted including use of insecticides, pesticides, herbicides and also fungicides [1,2]. These chemicals come in contact with fish directly after spraying or by rainfall through runoff during monsoon. Contamination of water by pesticides either directly or indirectly may severely affect the normal physiology, biology and early development of aquatic organisms that can lead to fish kills or reduced fish productivity [3,4].

Among different used pesticides, sumithion, O, O Dimethyl O-(3- methyl-4-nitrophenyl) having fenitrothion 500 g/kg as active ingredient, is widely used in Bangladesh. It is effective to control a wide range of important insects and certain other arthropod pests. It is mainly used to control beetles in paddy fields. It is also used in fish nursery ponds to control tiger bugs (*Cicindela* spp.). Since sumithion is widely used for crop protection and for eradication of aquatic insects in fish ponds, ultimately, the surface and ground water might be highly contaminated due to this agricultural runoff pesticide [5,6]. Sumithion is considered somewhat toxic to fish [7]. The pesticide affects the aquatic ecosystem by interrupting the aquatic food chain resulting in the loss/shift in abundance of natural invertebrate and vertebrate species in the aquatic environment [8,9]. It has been reported that several organophosphate pesticides, such as malathion altered histopathology and molecular disorder of liver and kidney in mice [10], triazophos and deltamethrin inhibited AChE activity in *Channa punctatus* [11] and imidacloprid caused the histopathological changes, activation of TNF-α, iNOS, 8-OHdG biomarkers, and alteration of caspase3, iNOS, CYP1A, MT1 gene expression levels in common carp [12]. Therefore, since sumithion is widely used for crop protection and for eradication of aquatic insects in fish ponds, it is very important to know the extent of damage being done by this chemical to fish and other aquatic lives.

Fish is very vulnerable to the changes in different water quality parameters which might be directly imitated in their blood parameters [13,14]. Blood parameters are considered as essential indicators to physiological stress caused by any internal or external deviations that affect homeostasis in fish [[15], [16], [17], [18], [19]]. Micronucleus (MN) is a small mass of cytoplasmic chromatin present outside of the central nucleus which is made during the nuclear division of the acentric chromosome fragments [20,21]. The formation of micronucleus in the erythrocyte assay has been used to examine the stress caused by different pollutants [14,[22], [23], [24]]. Similarly, assessment of nuclear and cellular abnormalities of erythrocytes is also a very important analytical technique to assess the stress caused by any environmental contaminants [25,26].

Striped catfish (*Pangasianodon hypophthalmus*), popularly known as Thai pangas, is an exotic fish in Bangladesh introduced from Thailand in 1990 [27]. The culture of this species contributes significantly in the annual fish production as well as livelihood of the rural people of the country. Since its inception, monoculture of the fish has widely been practiced. The culture of this species expanded rapidly due to its fast growth rate, tolerance of wide range of environmental conditions, grow well in high stocking density, easy rearing and seed production, high consumer demand, advantage of long distance transportation in live condition and farmers` opportunity to get higher economic gain than in culturing some other species [27]. Moreover, studies has been reported that polyculture of striped catfish with planktivorous carp reduce excessive phytoplankton growth, improve water quality, increase fish production and economic return [[28], [29], [30], [31]]. A total of about 70,000 farmers are involved in pangas farming covering about 43,000 ha land areas in Bangladesh. In 2017-18 this fish species contributed about 11% (0.45 million MT) of the annual total fish production of Bangladesh [32]. There are some studies on toxicity of sumithion on different fish species [[17], [18], [19],[33], [34], [35]], but attempt has not been made to know the toxicity of this insecticide on this important fish. Considering the importance, the present study was aimed to assess the toxicity of sumithion on striped catfish.

## 2 Materials and methods

### 2.1 Experimental fish

Striped catfish, *Pangasianodon hypophthalmus* was selected for the experiment. Forty five (45) days old healthy and active fingerlings of striped catfish were procured from a local fish farm. The mean length and weight of the fishes were 13.11 ± 0.61 cm and 14.34 ± 2.31 g, respectively. Before start of the experiment, the fingerlings were reared in aquaria at 25 ± 0.5 °C under natural photo-regimen about (12/12 h, light/dark) for a period of 21 days. Commercial fish feed (Popular Poultry & Fish FeedsLtd., Bangladesh) containing 35% crude protein was applied at the rate of 3–5% of the body weight of the fish twice a day in the morning and in the afternoon.

### 2.2 Procurement of the pesticide

Agriculture grade organophosphorus pesticide compound, sumithion in original sealed container was procured from an authorized dealer in Mymensingh, Bangladesh. It was in liquid form and white in colour. The expiry date of the test pesticide was checked prior to start of the experiment.

### 2.3 Experimental design and procedure to determine lethal concentration of sumithion

A static acute toxicity bioassay was performed according to standard method to determine the median lethal concentration (LC50) of sumithion for striped catfish fingerlings. Ten fingerlings were stocked in each cleaned glass aquarium (75 cm × 45 cm × 45 cm) filled with 30 l of tap water. Adequate aeration was maintained throughout the experimental period. The fishes were exposed to six (0 mg/l as control, 3 mg/l, 4 mg/l, 5 mg/l, 6 mg/l and 7 mg/l) concentrations of sumithion each with three replications. The application of the pesticide was repeated at every 24 h with a regular total exchange of water. Records of mortality were made at logarithmic time intervals (24, 48, 72, and 96 h) from the beginning of the test. Several inspections were made during the experimental period at every 12 h and dead fishes were removed immediately. A fish was considered as dead when respiratory movement of the opercula stopped and there was no response to touch.

### 2.4 Blood sampling

After 96 h of exposure, blood was collected from the survived fishes. The fishes were carefully collected and immediately anesthetized with clove oil (5 mg/l). After cutting the caudal peduncle, blood samples were collected and pushed into a sterilized centrifuge tube containing anticoagulant (20 mM EDTA). It took less than one minute per fish to complete the blood withdrawal process, which was deemed important to prevent stress impacts to minimize any mistake in normal blood values.

### 2.5 Measurement of hemoglobin (Hb)

Hb (%) was measured using a SAHLI’s hemometer (Model-3243000, MARIENFELD, Germany). At first 90 μl 0.1 N HCl was taken in an Eppendorf tube using micropipette. Then 10 μl of blood was added and the tube was shaked thoroughly for proper mixing. After 2–3 min the mixture was transferred to the tube of the hemometer. Then distilled water was added in drops until the color was adjusted with the colorimeter of the hemometer. When the color was adjusted then the reading was taken up to the level of the mixture specified on the body of the tube.

### 2.6 Estimation of the number of red blood cells (RBCs)

To count RBCs, 995 μl RBC diluting fluid (Hayem’s fluid) was taken in an Eppendorf tube. Then 5 μl blood was added with the fluid. During counting, 10 μl of RBC solution and a small amount of Giemsa stain was taken on a haemocytometer. After covering by a cover slip, it was observed under a light microscope. During counting, 5 large square units (each large square contains 16 small square units) were selected randomly. Numbers of RBC within a large square unit (avoiding those touched any lines) were counted. Using this procedure, the number of RBC was counted from randomly selected 5 large square units. Then the total number of RBC was counted using the following formula.

$$\text{Number of red blood cells}(\text{RBCs})=\left(\frac{\text{sum of RBC}\times \text{ 4000}\times \text{ 200}}{5\times 16}\right)\text{cells/m}{\text{m}}^{3}$$

### 2.7 Estimation of the number of white blood cells (WBCs)

To count WBCs, 195 μl WBC diluting fluid (Turk’s fluid) was taken in an Eppendorf tube. Then 5 μl blood was added with the fluid. During counting, 10 μl of WBC solution and a small amount of Giemsa stain was taken on a haemocytometer. After covering by a cover slip, it was observed under a light microscope. In case of WBC, the total number of WBC found within large squares of four corners were counted. Then the total number of WBC was counted using the following formula.

$$\text{Number of white blood cells}\left(\text{WBCs}\right)=\left(\frac{\text{sum of WBC}\;\times \;40}{0.1}\right)\text{cells/m}{\text{m}}^{3}$$

### 2.8 Measurement of the blood glucose level (mg/dl)

Blood glucose level (mg/dl) was measured using glucose strips in a digital EasyMate® GHb, blood glucose/hemoglobin dual-function monitoring system (Model: ET- 232, Bioptik Technology Inc. Taiwan 35057).

### 2.9 Analysis of frequencies of formation of micronucleus (MN)

Blood was smeared on clean glass slides and air dried for 10 min. The smear was stained with 5% Giemsa after fixation in methanol for 10 min. The slides were rinsed with distilled water and air dried overnight, and mounted with DPX. The MN was observed under a light microscope (MICROS MCX100LED, Austria) which was connected to a video camera (AmScope 1000). Three slides were prepared from each fish blood and two thousand cells from each slide were scored. Only cells have been scored with intact cell and nuclear membrane. The blind scoring of MN was conducted on randomized coded slides to minimize the technical variety. The MN was separated from or marginally overlapped with the primary nucleus as long as the nuclear border was clearly identified and MN was similarly stained as the primary nucleus [36].

### 2.10 Monitoring of water quality parameters

The dissolved oxygen, free CO_2_, pH and total alkalinity of water of each aquarium were measured during the experimental period. Dissolved oxygen (DO) was estimated by a DO meter (Model DO5509, Lutron, made in Taiwan). The pH of water was determined by a portable pH meter (Model number- RI 02895, HANNA Instruments Co.). The free CO_2_ of water was determined by titrimetric method using phenolphthalein indicator and 0.0227 N NaOH titrant. Total alkalinity of water was determined by titrimetric method using methyl orange indicator and 0.02 N H_2_SO_4_ titrant.

### 2.11 Data analysis

Values are expressed as means ± standard deviation (SD). To test the statistically significant difference among the different concentrations of sumithion, one-way analysis of variance (ANOVA) was carried out followed by Tukey's post hoc test. Statistical significance was set at p < 0.05. Statistical analyses were performed using PASW Statistics 18.0 software (IBM SPSS Statistics, IBM, Chicago, USA).

## 3 Results

### 3.1 Lethal concentration value of sumithion for the striped catfish

**Fig. 1:**
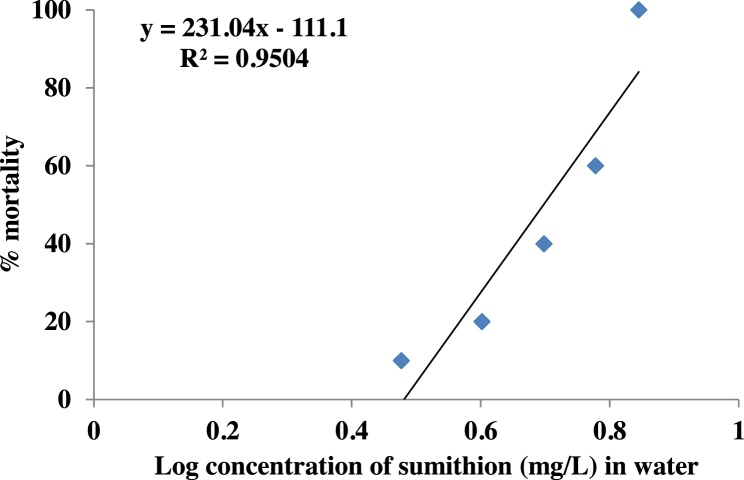
Linear transformation and the relationship of Probit of log concentration of sumithion used to determine LC_50_ value.

The lethal concentration of sumithion for the striped catfish was determined at sumithion level ranged from 3 to 7 mg/l. There was no mortality at control (0 mg/l) during 96 h exposure period. Percentage mortality of fish in different concentrations of sumithion is shown in Table 1. The probit analysis on number of observed dead fishes was performed after 96 h exposure at different concentrations of sumithion. Probit analysis showed that the median lethal concentration that is the concentration for 50% mortality of the fishes was 5.886 ppm. The linear transformation of the percentage mortality against the log concentration of sumithion is shown in Fig. 1.

**Table 1:** Mortality percentages of fish exposed to sumithion 0–7 mg/l of water.

Concentrations of sumithion (mg/l)	Initial no. of fish	Cumulative count of dead fish with time of exposure				% of mortality
		24 h	48 h	72 h	96 h	
0	30	0	0	0	0	0
3	30	0	0	0	3	10
4	30	0	0	0	6	20
5	30	0	0	0	12	40
6	30	3	3	15	18	60
7	30	15	15	30	30	100

### 3.2 Effects of sumithion on the blood hemoglobin (Hb)

**Fig. 2:**
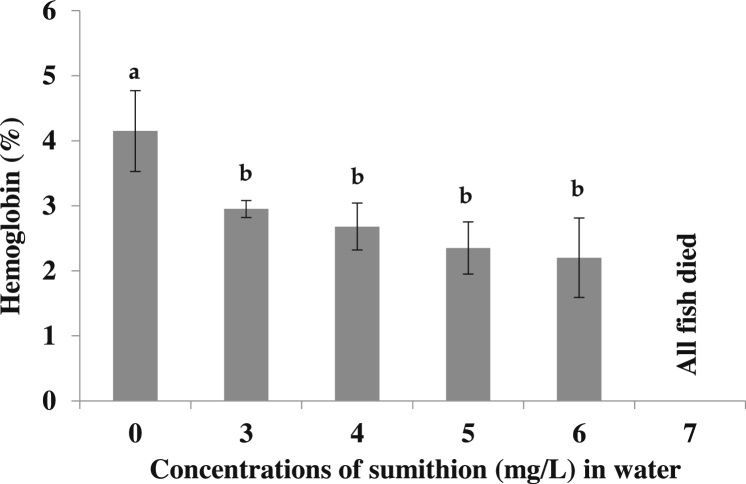
Changes in the hemoglobin (%) of *P. hypophthalmus* exposed to different sumithion concentrations for a period of 96 h. Values represent the mean ± SD (n = 4).

The values of the blood Hb level of the experimental fishes were examined after exposure of the fishes to different sumithion concentrations. Percentage of the Hb level were found to be decreased significantly (p < 0.05) with the increase of the toxicity of sumithion at 96 h of exposure period in the concentrations of 3–6 mg/l compared to control (0 mg/l), while at 7 mg/l all the fishes died within 72 h exposure (Fig. 2).

### 3.3 Effects of sumithion on the red blood cells (RBCs)

**Fig. 3:**
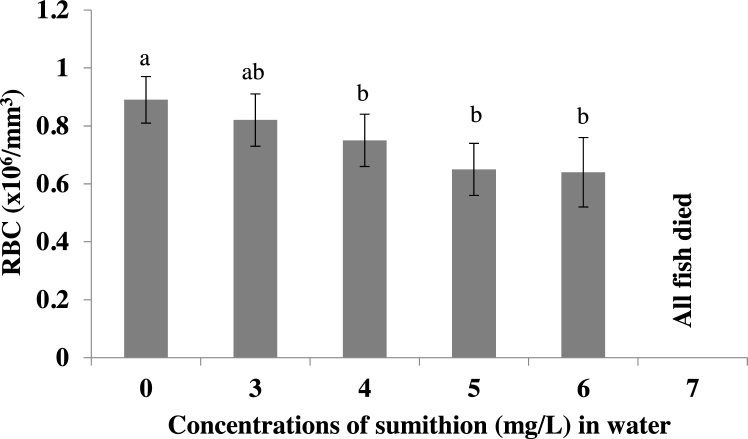
Changes in the RBC (cells x 10^6^/mm^3^) of *P. hypophthalmus* exposed to different sumithion concentrations for a period of 96 h. Values represent the mean ± SD (n = 4).

Red blood cells, or erythrocytes, are the most common type of blood cell which is the principal means of delivering oxygen to the body tissues via the blood flow through the circulatory system in vertebrates. Similar to Hb, RBCs count (×10^6^/mm^3^) was found to be decreased significantly (p < 0.05) in higher concentrations of sumithion at 96 h of exposure (Fig. 3).

### 3.4 Effects of sumithion on the white blood cells (WBCs)

**Fig. 4:**
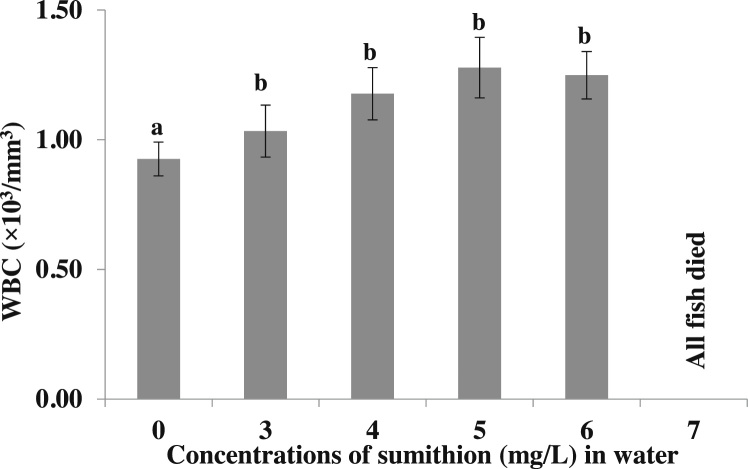
Changes in the WBCs (cells x 10^4^/mm^3^) of *P. hypophthalmus* exposed to different sumithion concentrations for a period of 96 h. Values represent the mean ± SD (n = 4).

White blood cells (WBCs), also called leucocytes, are the cells of the immune system those are involved in defending the body against both infectious diseases and foreign materials. The WBC was significantly (p < 0.05) increased in higher concentrations of sumithion at 96 h of exposure (Fig. 4).

### 3.5 Effects of sumithion on the blood glucose level (mg/dl)

**Fig. 5:**
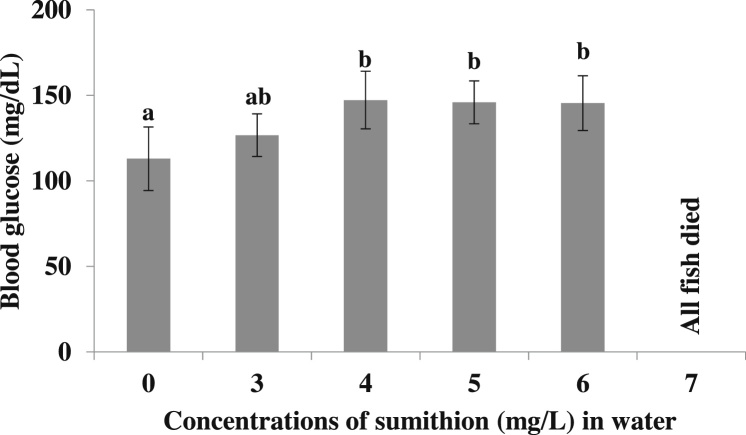
Changes in blood glucose level (mg/dl) of *P. hypophthalmus* exposed to different sumithion concentrations for a period of 96 h. Values represent the mean ± SD (n = 4).

The blood glucose levels of the experimental fish were examined after exposure of fish to sumithion. The blood glucose levels were significantly (p < 0.05) increased with the toxicity of sumithion at 96 h of exposure period in concentrations of 3–6 mg/l compared to control (0 mg/l), whereas all the stocked fishes died at 7 mg/l (Fig. 5).

### 3.6 Formation of micronuclei (MN) induced by sumithion

**Fig. 6:**
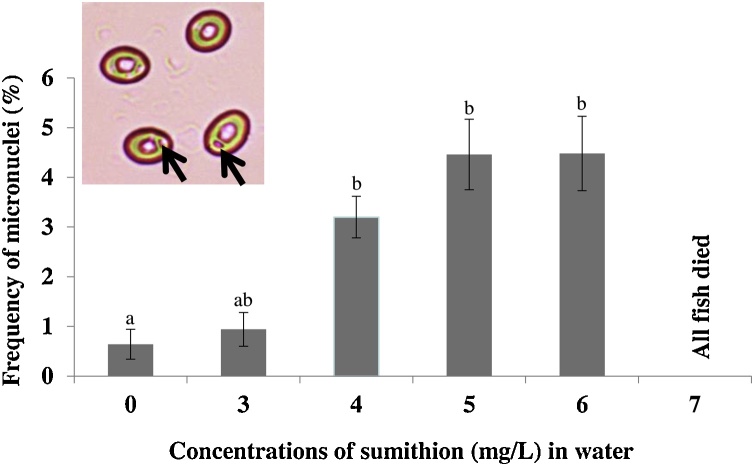
Frequency of micronuclei (MN) in the erythrocytes of Thai pangas exposed to different concentrations of sumithion. Values with different alphabetical superscripts differ significantly (p < 0.05) among concentrations within duration in MN. All values are expressed as mean ± SD (n=3). Three slides were prepared from blood of each fish and 2000 cells were scored from each slide. MN is shown by an arrow.

A statistically significant (p < 0.05) increase in the frequency of MN was noted in fishes exposed to sumithion concentrations of 3–6 mg/l compared to control (0 mg/l). All fishes died at 7 mg/l. About three to four folds increase in the frequency of MN noted at higher concentrations indicated the genotoxic effects of the sumithion (Fig. 6).

### 3.7 Water quality parameters

Water quality parameters play an important role in the growth and development of aquatic organisms. Some of the important water quality parameters (dissolved oxygen, free CO_2_, pH and total alkalinity) were measured during the study period (Table 2). Dissolved oxygen significantly decreased and free CO_2_ significantly increased with the increase of the concentrations of sumithion (Table 2). The values of pH and total alkalinity were almost uniform during the study period (Table 2).

**Table 2:** Water quality parameters during the study period.

Water quality parameters	Concentration of sumithion (mg/l)					
	0	3	4	5	6	7
Dissolved Oxygen (mg/l)	6.96 ± 0.85^a^	5.73 ± 0.75^b^	5.63 ± 0.21^b^	5.16 ± 0.75^b^	5.50 ± 0.52^b^	5.83 ± 0.21^b^
Free CO_2_ (mg/l)	4.00 ± 0.00^a^	8.00 ± 2.00^b^	8.67 ± 1.02^b^	08.66 ± 2.00^b^	9.33 ± 2.31^b^	9.33 ± 2.31^b^
pH	8.43 ± 0.06	8.16 ± 0.06	8.23 ± 0.06	8.30 ± 0.52	8.40 ± 0.10	8.70 ± 0.06
Total alkalinity (mg/l)	156.0 ± 4.6	152.6 ± 9.2	190.6 ± 7.5	173.3 ± 6.2	180.0 ± 3.0	180.0 ± 8.1

Values of a single water quality parameter in a row with different alphabetical superscripts are significantly (p < 0.05) different. All values expressed as mean ± SD.

## 4 Discussion

A number of studies have been reported on the differential acute toxicity of sumithion for several fish species. The 96 h LC50 value (5.9 mg/l) of sumithion for striped catfish found in the present study is less than the values of 8.1 for common carp [17], 11.8 mg/l for *Heteropneustes fossilis* [37] and 15.3 mg/l for *Gila elegance* [38]. In contrast, lower 96 h LC_50_ values were found in brook trout (1.7 mg/l), bluegill sunfish (3.8 mg/l) and *Oreochromis niloticus* (2.2 mg/l) [39]. The variation of acute toxicity of any chemicals depends on the physiological conditions of the concerned species, their habitat and chemical purity of the used chemicals and some water quality factors especially dissolved oxygen, pH etc. [40].

Hemoglobin (Hb) is the iron-containing oxygen-transport metalloprotein in the red blood cells which carries oxygen from the respiratory organs (lungs or gills) to the rest of the body (i.e. the tissues) where it releases the oxygen to burn nutrients to provide energy to power the functions of the organism in the process called metabolism. In the present study, Hb content in the blood of the fish exposed to the different concentrations of sumithion decreased significantly. A similar decreased value of Hb was also reported in common carp exposed to sumithion [17] and malathion [18]. The observed decrease in hemoglobin levels in striped catfish in the present study may be due to the disruptive action of the pesticides on the erythropoietic tissue as a result of which the viability of the cells might be affected. Similar to Hb, the number of RBCs was found to be decreased in fishes subjected to different concentrations of sumithion, also might be because of failing of hematopoietic system. Similar to the present results, a decrease in the number of RBC was reported in rainbow trout exposed to diazinon [41] and in *Clarias gariepinus* exposed to lead nitrate [42]. Inhibition of erythropoiesis and increase in the rate of erythrocyte destruction in hematopoietic organs is the cause of decrease in RBC count [43]. In the present study, the significant decrease of RBC content might have resulted from the oxygen deficiency in the body or from the lowering of the oxygen content of the water due to the presence of sumithion.

In the present study, WBC significantly increased with the increase of the toxicity of sumithion at 96 h of exposure period compared to control. This is may be due to the leucocytosis under chemical stress, deemed an adaptive value to the tissue. This also helps to remove necrosed tissue cell debris at a faster rate [44]. The immediate stimulation of immunological defense may result in leucocytosis in fish in the presence of foreign particles or under pathological conditions [44]. In the presence of foreign particles or under pathological circumstances, leucocytosis in fish may be the result of direct stimulation of immunological defense [44]. The rise in the number of WBCs can be linked with rise in the manufacturing of antibodies, which helps in the survival and regeneration of malathion-exposed fish [43].

In the present investigation, blood glucose was found to be increased significantly with gradual increase of sumithion concentrations. Increase of the amount of blood glucose in fish demonstrates the stressed situation of the fish when exposed to pesticides. Increased concentrations of glucose may be trigger hyperglycemic condition due the reaction of the hormone caused by stress. Such elevation may be due to the increased reaction of stressed fish to gluconeogenesis to meet their additional energy requirements [45]. There have been reports of changes in blood glucose concentrations in *H. Fossilis* subjected to testosterone sub-lethal concentration [46]. Hypoglycemia was noted in *H. Fossilis* exposed to a variety of pesticide concentrations such as rogor and aldrin [47]. This is likely owing to the fast use of blood glucose during hyper excitability, shocks and tremors, characteristic behavior of fish toxicity to organophosphate pesticides [48].

Blood is considered as the pathophysiological reflector of the body and therefore, blood cells like erythrocytes are important in diagnosing the functional and structural position of fish exposed to toxicants. Erythrocytes are capable to respond to a few environmental obsesses and alterations of erythrocyte (cellular and nuclear) represent the most common reflection towards pesticides present in water bodies [49]. In the present study, there was a significant increase in the frequency of formation of micronucleus (MN). Similar increases in the frequency of formation of MN were observed in *Oreochromis mossambicus* [50] and *Channa punctatus* [51] due to arsenic exposure, and in *Barbonynus gonionotus* [14] due to convoy (a quinalphos containing insecticide) exposure. The increase in the formation of MN in the present study in striped catfish due to pesticidal toxicity of crop insecticide sumithion is identified as a good genotoxic biomarker for monitoring the impact of agricultural pesticide in the environment.

The findings of the present research will help the policy makers to make people conscious about the impact of indiscriminate use of insecticides in crop fields on normal physiological development of fish and other aquatic organisms. Moreover the research findings will help to find out a safety level of using this pesticide in crop lands through further research.

## Declaration of Competing Interest

The authors have no conflict of interests. The authors themselves are responsible for the content of the paper.
